# Aboriginal Populations and Their Neglected Tropical Diseases

**DOI:** 10.1371/journal.pntd.0002286

**Published:** 2014-01-30

**Authors:** Peter J. Hotez

**Affiliations:** 1 Departments of Pediatrics and Molecular Virology and Microbiology, National School of Tropical Medicine at Baylor College of Medicine, Houston, Texas, United States of America; 2 Sabin Vaccine Institute and Texas Children's Hospital Center for Vaccine Development, Houston, Texas, United States of America; 3 James A. Baker III Institute for Public Policy, Rice University, Houston, Texas, United States of America

*Although Aboriginal people make up a small percentage of the worlds population, they are disproportionately affected by poverty and neglected tropical diseases (NTDs). Unless prioritized, Aboriginal populations may be the last to receive access to essential medicines as part of global NTD elimination efforts.*

An estimated 370 million people are today classified as belonging to Indigenous or Aboriginal groups [1], [2]. According to Gracey and King, included among the major criteria defining these populations are self-identification as belonging to an Indigenous group historical ties to specified geographic areas and natural resources, frequently followed by external invasion or colonization a distinct culture with beliefs and ceremonies and resolve to maintain ancestral environments and manage their own affairs [1]. However, there is not universal agreement on what constitutes an Aboriginal group, leading in some cases to exclusion or further marginalization [1]. As a whole, Aboriginal populations are disproportionately impoverished, accounting for 15 of global poverty even though they comprise only 5 of the global population [3]. Poverty, especially rural poverty, and its associated poor housing and sanitation, environmental degradation, inadequate or improper nutrition, forced migrations, and lack of access to health care, combine and synergize to create a number of adverse health consequences for Aboriginal populations [1], [2]. These include a spectrum of non-communicable diseases such as diabetes, obesity, hypertension, and cardiovascular disease (frequently related to tobacco consumption), as well as interpersonal violence and suicide often linked to alcohol and drugs [1], [2].

Aboriginal populations are also disproportionately affected by infectious diseases, especially infections of the skin (e.g., impetigo and tungiasis), eye (e.g., trachoma), and ear (e.g., acute and chronic otitis), dental caries, respiratory and urinary tract infections, and diarrheal diseases [1]. Neglected tropical diseases (NTDs) are especially common among Aboriginal populations. Shown in Table 1 are five representative groups of Aboriginal peoples and their major NTDs. The most common NTDs are intestinal helminth and protozoan infections zoonotic parasitic infections neglected bacterial infections such as trachoma, leprosy, and yaws and a number of important vector-borne illnesses, including malaria and dengue. Many, but not all, are included in the list of 17 NTDs as defined by the World Health Organization [4]; others are on the expanded list of diseases as defined by *PLOS Neglected Tropical Diseases* [5].

**Table 1:** Major Aboriginal populations and their neglected tropical diseases (NTDs).

Indigenous People	Location/Origin	Approximate Population	Major NTDs	Ref.
Orang Asli	Malaysian Peninsula	150,000^a^^,^ b	Soil-transmitted helminth (STH) infections, intestinal protozoan infections, malaria, dengue	[6]–[22]
Australian Aboriginals	Australia: Northern Territory and elsewhere	500,000^c^–600,000^d^	Strongyloidiasis and other STH infections, Melioidosis, Trachoma, Scabies and secondary pyoderma	[23]–[32]
Yanomami	Amazon rainforest: Brazil and Venezuela	–20,00030,000^e^^,^ f	Onchocerciasis, STH infections, Tungiasis, Trachoma, Malaria	[36]–[40]
Inuit	Arctic Circle: Canada, Alaska, Greenland, Russia^d^	150,000^g^^,^ h	Zoonotic helminth infections (Echinococcosis, Trichinellosis), Giardiasis, Toxoplasmosis	[41]
African Pygmy (Bayaka)	Central African rainforest: Cameroon, Central African Republic, Congo, Equatorial Guinea, Gabon^e^	–300,000500,000^i^	STH infections, Scabies, Leprosy, Yaws, Malaria	[42], [43]

a http://en.wikipedia.org/wiki/Orang_Asli.

b Malaysia: Primary Health Care Key to Intersectoral Action for Health and Equity. WHO 30 Aug 2007: http://www.who.int/social_determinants/resources/isa_primary_care_mys.pdf.

c http://en.wikipedia.org/wiki/Aboriginal_Australians.

d Australian Bureau of Statistics. http://www.abs.gov.au/AUSSTATS/abs.nsf/Latestproducts/946D4BC28DB92E1BCA25762A001CBF38?opendocument. Accessed 2 April 2013.

e http://en.wikipedia.org/wiki/Yanomami.

f –WHO Weekly epidemiological record. 18 September 2009, No. 38, volume 84. p. 385396. http://www.who.int/wer/2009/wer8438/en/index.html.

g http://en.wikipedia.org/wiki/Inuit.

h *Encyclopedia of the Arctic*, Routledge (2005), Mark Nutall, editor.

i Reference [42].

## Asian and Oceanic Aboriginal Populations

The Asia-Pacific region hosts the largest concentration of Aboriginal peoples, accounting for 70 of the global Aboriginal population [3]. Although there is no comprehensive survey of the Indigenous peoples in the Asia-Pacific region and their diseases, the Orang Asli of peninsular Malaysia and the Aboriginal Australians have undergone intensive study over the past decade with respect to their NTDs and other infections.

### Orang Asli (Malaysia)

The term Orang Asli applies to more than a dozen Indigenous groups, comprising a population of approximately 150,000 people living in peninsular Malaysia. Although Malaysia has sustained high levels of economic growth over the last few decades, the growth has not always included Malaysias rural population, especially the Orang Asli [6–21]. Among the major NTDs affecting these groups are high prevalence and intensity rates of hookworm infection and other soil-transmitted helminthiases [6–9], which have been identified as key determinants of malnutrition and poor school performance and attendance among Malaysian schoolchildren [10], [11]. Additional intestinal parasitic infections common to the Orang Asli and other rural Malaysian populations are amoebiasis (including an unusual form caused by *Entamoeba moshkovskii*) [12], [13], cryptosporidiosis [14], and giardiasis [15]. Toxoplasmosis is also widespread [16]. Malaria from *Plasmodium falciparum* infection is common among the Orang Asli and an important cause of morbidity and mortality [17–18], and an unusual malaria species, *Plasmodium knowlesi*, is endemic [19–21]. In contrast, dengue and other arboviral infections have been primarily urban diseases, but it has been noted that rural infections are becoming increasingly common [22].

### Australian Aboriginal Populations

Australia hosts a large Aboriginal population of approximately 0.5 million people. New South Wales and Queensland are the states with the largest overall population, but the Northern Territories has the highest percentage of Aboriginal people compared to other states. Significant numbers of Australias Aboriginal population (approximately one-third) now also live in major cities. Some of the major NTDs affecting the impoverished populations of Oceania, including Australias Aboriginal population, were reviewed previously [23]. Briefly, the NTDs predominate in remote rural areas and include a significant problem resulting from strongyloidiasis, which is hyperendemic among this population and often associated with human T-lymphotropic virus 1 (HTLV-1) co-infection [24], [25]. Hookworm caused by *Ancylostoma duodenale* is also widespread, as are intestinal protozoan infections [23], [26]. Skin and eye NTDs are major public health threats. An interesting pathologic sequence, typically initiated by ectoparasitic scabies, followed by secondary group A streptococcal (GAS) infections leading to impetigo (also known as pyoderma), has been elucidated and found to be extremely common (prevalence often exceeding 50) among both adult and pediatric Aboriginal populations in Australia [27], [28]. The scabies-GAS co-infections are also leading to high rates of post-streptococcal sequelae, including glomerulonephritis and rheumatic heart disease [27], [28]. In some cases secondary staphylococcal infections occur, including those caused by methicillin-resistant *Staphylococcus aureus* (MRSA) [29]. Several attempts to implement mass treatment for scabies among Australias aboriginal population (using either ivermectin or topical permethrin) have been attempted to interrupt this sequence [27], [28]. Trachoma is also an important NTD among this population, with blinding trachoma still common in remote rural areas of Australia, although recently the Australian government committed to eliminating this disease [30], [31]. Melioidosis (*Burholderia pseudomallei*' infection) is also common during the wet season among Australias Aboriginal population [32].

## Aboriginal Populations in the Americas

According to the Pan American Health Organization there are an estimated 40 million Indigenous people in the Latin American and Caribbean region (approximately 7 of the total population), with the nations of Peru, Guatemala, Bolivia, and Ecuador having the highest percentage of Indigenous populations compared to other countries [33, 34]. In many instances, American Indigenous groups are found disproportionately on plantations and agricultural labor camps where they are exposed to high rates of vector-borne NTDs [33–35]. The Aboriginal populations of the Americas are infected with a variety of NTDs, often depending on their geographic location, for example, whether they are in mountainous areas or tropical lowlands [33–36]. Two examples are provided here.

### Yanomami (Brazil and Venezuela)

The Yanomami are a group of more than 10,000 extremely isolated rainforest people living in large communal houses in two Amazonian states of Brazil (Roraima and Amazonas) and in neighboring Venezuela [36]. Just like the Asian and Oceanic Aboriginal people, the Yanomami suffer from high rates of intestinal helminth infections, as well as skin and eye infections [36]. They are also vulnerable to two important mycobacterial infections, tuberculosis and leprosy [36]. A major skin infection is tungiasis as a result of the ectoparasitic flea, *Tunga penetrans*, which may be transmitted from dogs often living within communal houses [36]. Two important eye infections are onchocerciasis and trachoma [37–39]. The rainforest area between Brazil and Venezuela represents one of six remaining foci for endemic onchocerciasis in the Americas [37], [38]. It represents a challenging area for purposes of onchocerciasis elimination because of its size and the fact that the Yanomami are widely dispersed [37]. The current approach to onchocerciasis elimination in the region relies on twice-yearly mass treatments with ivermectin, which so far has roughly cut in half the prevalence of this infection and caused a dramatic reduction in the infection intensity as measured by the number of microfilariae per milligram of skin [38]. However, there has not been a significant reduction in the prevalence when measured by palpable nodules or onchodermatitis [38]. High rates of trachoma are also present among Yanomamiaccording to one study an overall prevalence of 30 was recorded, with females significantly more affected than males [39]. Efforts have been undertaken in some areas to provide mass treatments with azithromycin [39]. Finally, it has been noted that the Yanomami suffer from high infection rates of malaria, as well as anemia [40]. The contribution of hookworm or other parasitic infections to anemia among the Yanomami has not been widely studied.

### Inuit (Arctic Canada, United States, Greenland, Russia)

The Inuit are comprised of Aboriginal peoples in adjacent areas of Canada, the United States (Alaska), Greenland, and eastern Russia. Their major NTDs were reviewed previously [41]. Briefly, despite subzero Arctic temperatures, the Inuit are vulnerable to a number of NTDs transmitted as parasitic zoonoses and often acquired through ingestion of meat from marine mammals (e.g., walruses and seals), polar bear, and caribou [41]. Major examples include trichinellosis caused by a unique species (*Trichinella nativa*) and associated with prolonged diarrhea, eosinophilia, and toxoplasmosis [41]. Echinococcosis (both cystic and alveolar forms) is also present, although some investigators suggest that the incidence has diminished as definitive dog hosts are gradually being replaced with mechanized vehicles [41].

## Aboriginal Populations in Africa

A large number of Indigenous peoples live in sub-Saharan Africa, including up to 500,000 Pygmy (also known as Bayaka) in the Central African rainforests of Cameroon, Central African Republic, Congo, Gabon, and elsewhere [42]. The basis of their short stature is unknown, although it may be related to genetic or epigenetic factors associated with growth hormone or growth hormone receptor [43]'. The health of Africas Aboriginal populations was reviewed previously, with emphasis on the Pygmy and the San [42]. It has been noted that skin infections such as yaws and leprosy are more prevalent among the Pygmy than other African rural populations, as well as intestinal parasitic infections [42]. Both onchocerciasis and loiasis are endemic to the Central African rainforest. Scabies and chiggers are also present, and these populations are also more vulnerable to filovirus infections [42]. Malaria is also widespread [42].

## Summary Statement and Recommendations

Although the worlds Aboriginal populations are vulnerable to and suffer from a variety of different and often unrelated NTDs, several themes emerge that suggest an opportunity for common approaches to disease control and elimination. Important among these is the concept of mass drug administration (MDA) for NTDs that might be specifically relevant to indigenous groups, including their neglected tropical skin diseases [44].

### Mass Drug Administration (MDA)

Ivermectin targets a number of invertebrate pathogens, including *Strongyloides stercoralis* and other intestinal helminths and *Onchocerca volvulus*. Thus, MDA with ivermectin would have important health benefits for Aboriginal populations in Australia affected by high rates of strongyloidiasis and also for two populations highly vulnerable to onchocerciasis and/or loiasis, namely the Yanomami and African Pygmy groups. Ivermectin could also have a significant impact on ectoparasitic infections, such as scabies or tungiasis, which are widespread in these populations and therefore have indirect benefits to combat secondary streptococcal infections and post-streptococcal sequelae [44]. Moving forward on MDA programs that use ivermectin may require additional safety data in pregnancy and small children.

'Equally important are the potential benefits of MDA with azithromycin, particularly for reducing trachoma rates among Australias Aboriginal populations and the Yanamami. There is also recent evidence for the beneficial effects of azithromycin MDA on reducing the prevalence of yaws [44], [45], which could also effect a significant health improvement among multiple Aboriginal populations in Australia and among the African Pygmy populations. Earlier studies have shown that MDA with azithromycin could also reduce overall mortality in Ethiopia and possibly elsewhere [46].

### Providing Access to Essential Medicines and Other Control Opportunities

Overall, the worlds Aboriginal populations may lack access to essential medicines for NTDs used for mass treatments, including ivermectin and azithromycin as outlined above, as well as deworming medicines for intestinal helminth infections and schistosomiasis. For instance, the benefits of MDA with albendazole for the control of hookworm among Aboriginal populations in the Northern Territories were recently reported [47]. As we move towards expanding global coverage for MDA of essential medicines, i.e., albendazole or mebendazole, praziquantel, ivermectin or diethylcarbmazine citrate, and azithromycin, with projected elimination targets as per the 2012 London Declaration for NTDs [48], we need to be cognizant of the worlds 300400 million Aboriginal peoples, many of whom are particularly vulnerable to these conditions. Given their history of lack of access to health care [1], [2], we risk the real possibility that these populations will also be among the last to receive essential NTD medicines. Unless we act now, they could represent the groups last to benefit from potential NTD elimination endgames.

MDA is not the solitary approach to controlling and eliminating NTDs. Such measures are a key component of more broad and encompassing measures that include sanitation, access to clean water, good food, integrated vector control and management, childhood immunizations, and personal and family hygiene [1], [2]. Ultimately, equal or even greater attention must be given to the key underlying social determinants of illness among the worlds Aboriginal populations, including improvements in housing and advances in overall economic development [1], [2]. In some cases, failure to address critical social factors can lead to poor compliance and adversely affect the sustainability of MDA programs [27]. In 2007, the United Nations (UN) Declaration on the Rights of the Indigenous Peoples was adopted by the UN General Assembly [3]. We need to recognize and prioritize the importance of NTD control and elimination measures as an essential element of that action.
